# Breaking the Wheel, Credibility, and Hermeneutical Injustice: A Response to Harris

**DOI:** 10.1007/s13347-024-00828-7

**Published:** 2024-11-29

**Authors:** Taylor Matthews

**Affiliations:** University of Southampton, Southampton, England

## Abstract

In this short paper, I respond to Keith Raymond Harris’ paper “Synthetic Media, The Wheel, and the Burden of Proof”. In particular, I examine his arguments against two prominent approaches employed to deal with synthetic media such as deepfakes and other GenAI content, namely, the “reactive” and “proactive” approaches. In the first part, I raise a worry about the problem Harris levels at the reactive approach, before providing a constructive way of expanding his worry regarding the proactive approach.

## Introduction

In his innovative article ‘Synthetic Media Detection, the Wheel, and the Burden of Proof’, Keith Raymond Harris critically examines two strategies that have been proposed in response to the development and popularisation of Generative Artificial Intelligence (GenAI) and so-called “synthetic media”. These are the *reactive* and *proactive* approaches. As Harris points out, each strategy has a number of benefits for dealing with the deceptive and sceptical implications of synthetic media. Despite this, he raises novel and interesting problems for each. In this commentary, I respond to Harris’ arguments. I first raise a worry about the effectiveness of his strategy against the reactive approach (§1), before offering a constructive way of developing his concerns about the proactive approach (§2).

1. **The Reactive Approach and the ‘Wheel Problem’**

Let me start with the reactive approach, which relies on algorithms to detect, distinguish, and label synthetic media from non-synthetic media. Although this approach looks promising initially, Harris claims that it faces a number of difficulties. Principal amongst these is what he terms the ‘wheel problem’ (pg. 7). In fact, he claims that this problem also arises for the *proactive approach*, but he later concedes that ‘a version of the proactive strategy holds promise for addressing’ it (pg. 10). For this reason, I shall focus solely on this problem as it relates to the reactive approach. What Harris calls the ‘wheel problem’ derives from a much older problem in epistemology known as The Problem of the Criterion. Roughly, this concerns what we know and how we can determine the criteria for knowledge. To determine what we know, it seems we need to have a criterion for distinguishing cases of knowledge. But to have this, it seems we also need to first know which cases count as knowledge. The problem arises because we cannot seemingly answer each question independently of the other, so we are stuck in a ‘wheel’, as it were (Chisholm, 1982: 62). Harris levels a novel, technological version of this problem at the reactive approach. Since the effectiveness of this approach depends on algorithms correctly identifying and distinguishing synthetic media from non-synthetic media, it relies on us being able to know that the media it identifies and distinguishes really is synthetic. But to know this, we need to know that the reactive approach applies the label “synthetic” reliably. And to know this, we need to know whether the media in question is synthetic or not. The problem, again, is that we cannot address one of these issues without addressing the other. This, he claims, casts doubt on the effectiveness of the reactive approach (pg. 7). Harris defends this claim by first arguing that simple reliabilism cannot help the reactive approach, comparing those who are guided by reliable algorithms to those who rely on clairvoyance to form their beliefs (Bonjour, 1980). Just as the latter’s beliefs are intuitively unjustified despite their reliability, so Harris claims that the beliefs of those who depend on reliable algorithms are also unjustified. This is because they have ‘no independent evidence for the reliability of that algorithm’ (pg. 7-8). As Harris acknowledges, though, is it not the case that ‘anyone relying on the algorithm would come to have evidence of its reliability’ (pg. 8)? If so, wouldn’t a track record of success here ‘vindicate the reliability of the algorithm over time’ (pg. 8)? Harris thinks not, claiming that ‘there is good reason to expect the performance of algorithms for detecting synthetic media to degrade over time, as technologies for producing synthetic media advance’ (pg. 8). Indeed, he claims that this is true ‘even if, as a matter of fact, the algorithm remains perfectly reliable’ (pg. 8). As a result, he concludes that one’s inferences about the reliability of the algorithm ‘would be unjustified’. Thus, the reactive approach fails to break the wheel. I find Harris’ appeal to the ‘wheel problem’ very interesting, but I worry that it is less effective in undermining the reactive approach than he thinks. While I agree with Harris that simple reliabilism cannot salvage the reactive approach, it does not follow from this that other, more sophisticated versions of reliabilism are useless. During peer-review, I suggested to Harris that *virtue reliabilism* could be a more promising alternative. On this family of views, justification or warrant does not attach to the reliability of a belief-forming process, but rather to an agent’s truth-conducive cognitive competences or abilities (Greco, 2010; Sosa, 2015). As I noted to Harris, our inductive reasoning is one such competence. When making inferences about the reliability of detection algorithms from their past success, an agent would not simply be forming their beliefs based on algorithms in the abstract, but rather doing so in full awareness that their inducive reasoning is generally a reliable cognitive faculty. In turn, they would acquire inductive evidence about the reliability of the algorithm from the antecedent reliability of their inductive reasoning. They would thus obtain the ‘independent evidence’ that Harris thinks is lacking with simple reliabilism. Not only would this make the agent’s inferences justified, but their beliefs would at least amount to what Sosa calls ‘animal knowledge’ (2007, 2015). So, while simple reliabilism might not be able salvage the reactive approach, virtue reliabilism looks far more promising in breaking the wheel. Harris acknowledges the merits of this suggestion in footnote 12 of the paper when he observes how one might defend the reactive approach by ‘insisting that competent induction from a track record of success is enough for a justified inference’ (pg. 8). However, ‘even if this this point is conceded’, Harris claims, the possibility of the algorithm degrading could still ‘compromise the safety and sensitivity’ of the beliefs based on the detection algorithm, thereby undermining their justification (pg. 8, fn. 12) (Pritchard, 2005; Nozick, 1981).The idea that degrading algorithms could comprise the safety or sensitivity of such beliefs was another strategy I suggested to Harris during peer-review, and it looks like a *prima facie* plausible response to my concern above. After all, if the algorithm degrades over time, then while one’s beliefs about an item of synthetic media might be true and competently formed in the actual world, it would be false in close modal possibilities because the algorithm’s reliability degrades. Thus, this belief would be unsafe, even if competently formed. Similarly, the agent’s inferential belief would also be insensitive because they would continue to falsely believe that the algorithm was reliable in close modal possibilities, when it no longer was. But if Harris is willing to concede the point about competent induction above, then his appeal to safety or sensitivity needs to effectively undermine the virtue reliabilist proposal above. The problem is that many virtue reliabilists think that neither safety nor sensitivity is necessary for knowledge or justification (Greco, 2010; Sosa, 2015). At best, Harris’ response undermines ‘impure’ virtue reliabilism that incorporates such conditions (Kelp, 2013; Pritchard, 2012). However, it does not undermine ‘pure’ virtue reliabilism. Since he concedes this point, Harris’ appeal to safety and sensitivity falls short of demonstrating that competent induction alone cannot salvage the reactive approach and break the wheel.Furthermore, Harris thinks an agent’s inferential beliefs would remain unjustified ‘even if, as a matter of fact, the algorithm remains perfectly reliable’ (pg. 7). Suppose we grant this. The problem, now, is that safety and sensitivity would play no role in Harris’ strategy. For notice that in every close modal possibility in which we infer the reliability of the algorithm from its past success, our beliefs would always turn out true because it is perfectly reliable. Consequently, there would never be a close modal possibility in which agents form false beliefs, and thus their beliefs would always be safe. Likewise, if the algorithm is perfectly reliable, then there would also be no close modal possibilities in which agents continue to infer the truth about the algorithm’s reliability, but their beliefs turn out false. Thus, their beliefs would also be sensitive. Accordingly, Harris’ appeal to safety and sensitivity fails to explain why our inferential beliefs in this stipulation would be unjustified. In conjunction with my proposal above, then, the reactive approach has more potential to break the ‘wheel’ than Harris gives it credit for.

2. **The Proactive Approach, Credibility, and Hermeneutical Injustice**

Although I have reservations about Harris’ concerns with the reactive approach, I think his argument against the *proactive approach* is more promising. The proactive approach deals with synthetic media by using blockchain and related technologies to verify non-synthetic media. In the process, it creates a ‘decentralised and immutable record of the provenance of pieces of media’ (pg. 5) (Laas, 2023). While Harris thinks that a version of this approach can potentially solve the ‘wheel problem’, he argues that it generates its own problem. Harris frames this problem in terms of epistemic injustice, claiming that the proactive approach threatens a ‘close cousin of testimonial injustice’ (pg. 10) (Fricker, 2007). In particular since it relies on agents using blockchain and other technology to verify non-synthetic media, Harris worries that the proactive approach risks creating scenarios whereby those less technologically-savvy or financially well-off are unable to access the technology to verify their non-synthetic media (pg. 10-11). In turn, those without this technology face the risk of having the credibility or ‘evidential weight’ of their non-synthetic media contributions unduly disvalued or discounted. So, even if the proactive approach goes some way towards restoring the evidential weight of video footage, Harris thinks, it would ‘not do so in an equalising way’ pg. 11).

I agree with Harris that the proactive approach threatens a form of epistemic injustice, and I think he convincingly dispels a worry that comes from appealing to this concept. Importantly, though, I think there are at least two further ways of developing his’ argument against the proactive approach on this score, both of which compliment his analysis. The first builds on Harris’ contention that the proactive approach risks deflating the credibility of certain peoples’ non-synthetic media contributions. This would certainly threaten a close cousin of testimonial injustice, conventionally understood. Another possibility, though, is that it risks conferring *too much* credibility on those with the means to verify their non-synthetic media contributions. Since Harris already appeals to the ‘distributive’ model of epistemic injustice (pg. 12, fn. 15) (Fricker, 2013; Coady, 2017), this suggestion would easily be accommodated by his argument. On the resulting view, the proactive approach would not just deflate the credibility of certain non-synthetic contributions, but it would simultaneously inflate the credibility of other such contributions. Not only would this honour the dynamic nature of epistemic injustices (Medina, 2013; Davis, 2016), but it would help illustrate Harris’ claim that the proactive approach risks producing ‘significant disparities in assignments of credibility’ (pg. 12).

My second suggestion develops on Harris’ contention that the epistemic injustice incurred by the proactive approach is ‘arguably a form of structural epistemic injustice (pg. 12) (Anderson, 2012). One way of cashing out this point is by appeal to *hermeneutical injustice*. As Fricker notes, this occurs when a significant area of one’s social experience is obscured from collective understanding because one has been marginalised from the practices that generate shared meanings and interpretative concepts (2007: 158). Notice that sharing and exchanging non-synthetic media is an important way of generating these “hermeneutical resources”. Yet if the credibility of certain peoples’ non-synthetic media contributions is unduly deflated, then the proactive approach plausibly excludes those agents from participating in such practices. In turn, it would risk placing these agents in a ‘hermeneutical lacuna’ (Fricker, 2007): rather than having their contributions correctly interpreted as “genuine” or non-synthetic, they would instead struggle to be taken seriously or dismissed as synthetic. Far from worrying whether the problem he identifies with the proactive approach falls ‘outside the boundaries of epistemic injustice’ (pg. 12), then, I think the suggestions above only reinforce Harris’ case for using this concept.

## Data Availability

Not applicable.
